# Identification of Nucleolin as a Novel AEG-1-Interacting Protein in Breast Cancer via Interactome Profiling

**DOI:** 10.3390/cancers13112842

**Published:** 2021-06-07

**Authors:** Seong-Jae Lee, Kyoung-Min Choi, Geul Bang, Seo-Gyu Park, Eun-Bi Kim, Jin-Woong Choi, Young-Ho Chung, Jinyoung Kim, Seok-Geun Lee, Eunjung Kim, Jae-Young Kim

**Affiliations:** 1 Graduate School of Analytical Science and Technology (GRAST), Chungnam National University, Daejeon 34134, Korea; seongjae.lee10@gmail.com (S.-J.L.); kyoungmin11@cnu.ac.kr (K.-M.C.); gue95@naver.com (S.-G.P.); kim.eunbi9795@gmail.com (E.-B.K.); 606480@naver.com (J.-W.C.); 2 Research Center for Bioconvergence Analysis, Korea Basic Science Institute (KBSI), Ochang 28119, Korea; bangree@kbsi.re.kr (G.B.); chungyh@kbsi.re.kr (Y.-H.C.); jinyoung@kbsi.re.kr (J.K.); 3 Bionanocomposite Research Center, Department of Science in Korean Medicine, Kyung Hee University, Seoul 02447, Korea; seokgeun@khu.ac.kr; 4 Natural Product Informatics Center, Korea Institute of Science and Technology (KIST), Gangneung 25451, Korea

**Keywords:** AEG-1, nucleolin, protein-protein interaction, breast cancer, metastasis, LC-MS/MS

## Abstract

**Simple Summary:**

Breast cancer is one of the most common cancers affecting women today. Astrocyte Elevated Gene-1 (AEG-1) is elevated in breast cancer patients and is associated with metastasis and poor prognosis. However, the mechanisms by which AEG-1 promotes breast cancer are not fully understood. This report focuses on a novel AEG-1 interacting protein, nucleolin (NCL), which we identified via mass spectrometry-based interactome profiling. We found NCL to be important for the oncogenic function of AEG-1 in breast cancer. Further, c-Met was identified as a novel mediator of the oncogenic function of the AEG-1-NCL complex. Collectively, our study suggests that targeting the AEG-1-NCL protein complex could be an effective therapeutic approach for the treatment of some breast cancers.

**Abstract:**

Breast cancer is one of the most common malignant diseases worldwide. Astrocyte elevated gene-1 (AEG-1) is upregulated in breast cancer and regulates breast cancer cell proliferation and invasion. However, the molecular mechanisms by which AEG-1 promotes breast cancer have yet to be fully elucidated. In order to delineate the function of AEG-1 in breast cancer development, we mapped the AEG-1 interactome via affinity purification followed by LC-MS/MS. We identified nucleolin (NCL) as a novel AEG-1 interacting protein, and co-immunoprecipitation experiments validated the interaction between AEG-1 and NCL in breast cancer cells. The silencing of NCL markedly reduced not only migration/invasion, but also the proliferation induced by the ectopic expression of AEG-1. Further, we found that the ectopic expression of AEG-1 induced the tyrosine phosphorylation of c-Met, and NCL knockdown markedly reduced this AEG-1 mediated phosphorylation. Taken together, our report identifies NCL as a novel mediator of the oncogenic function of AEG-1, and suggests that c-Met could be associated with the oncogenic function of the AEG-1-NCL complex in the context of breast cancer.

## 1. Introduction

Breast cancers remain not only the leading cause of cancer death, but also one of the most common cancers in females worldwide [1]. In 2020, the number of new breast cancer cases and deaths from breast cancer were reported to be 2.2 million and 684,996, respectively [2]. Treatment options for breast cancer include chemotherapy, hormone therapy, and radiation therapy [3], all of which have led to an improved 5-year survival rate for breast cancer patients. For example, in the United States, the 5-year survival rate is significantly higher for women with non-metastatic invasive cancers (90%) and patients with invasive breast cancer located only in the breast (84%) [4]. However, the 5-year survival rate for distant metastatic breast cancers drops to 20%, in part due to a lack of knowledge regarding the molecular mechanisms involved in metastatic breast cancers.

Astrocyte elevated gene-1 (AEG-1) [5], also known as metadherin (MTDH) [6] or lysine-rich CEACAM1 co-isolated (LYRIC) [7], was originally identified as a human immunodeficiency virus (HIV)-1-inducible transcript in primary human fetal astrocytes [8]. AEG-1 is markedly overexpressed in many solid tumors and functional studies have shown that AEG-1 promotes the proliferation, invasion, and metastasis of various cancer cells [9,10].

The oncogenic function of AEG-1 is known to be associated with multiple oncogenic signaling pathways. AEG-1 expression is reportedly induced by Ha-Ras-mediated PI3K-AKT signaling and its downstream mediator c-Myc [11]. AEG-1 promotes the AKT-mediated survival signaling pathway, suggesting that the Ras-PI3K-AKT pathway is closely linked to the oncogenic function of AEG-1 via the activation of a positive feedback mechanism [12]. AEG-1 regulates genes involved in the migration and invasion of cancer cells by activating the NF-κB pathway [13] and interacting with various oncoproteins [13,14,15,16,17]. AEG-1 interacts with AKT2, increasing cell survival and proliferation via phosphorylation of AKT2 and GSK3β [16]. Additionally, AEG-1 promotes the nuclear translocation of β-catenin via activation of the Wnt/β-catenin and ERK pathways by inducing LEF1 expression [18]. AEG-1 not only interacts with IKKβ to induce a decrease in IκBα in an IKKβ-dependent way [15], but also interacts with p65, a subunit of NF-κB, to induce the nuclear translocation of NF-κB [13].

In breast cancers, enhanced AEG-1 expression in clinical tissues has been discovered at both the mRNA and protein levels. Genomic amplification is one of the mechanisms by which AEG-1 is overexpressed in breast cancer [19,20,21,22], however, the underlying molecular mechanisms by which AEG-1 promotes breast cancer invasiveness have yet to be fully elucidated.

In this study, we mapped the AEG-1 interactome via affinity purification followed by LC-MS/MS analysis. We identified nucleolin (NCL) as a novel AEG-1 interacting protein and found that NCL is important for the oncogenic function of AEG-1 in breast cancer cells. Furthermore, we report that the AEG-1-NCL complex promotes c-Met activation, implying that NCL is likely a novel mediator of the oncogenic function of AEG-1 in the context of breast cancer.

## 2. Materials and Methods

### 2.1. Cell Culture

All cells were obtained from the American Type Culture Collection. 293T, HeLa and MDA-MB-231 cells were cultured in DMEM medium. BT549 and MCF-7 cells were cultured in RPMI1640 medium, and MCF-10A cells were cultured in DMEM/F12 medium. All culture media were supplemented with 10% fetal bovine serum (FBS) and an antibiotic-antimycotic (AA).

### 2.2. Transfection

Plasmid and siRNAs transfections were performed using Lipofectamine 3000 (Invitrogen, Carlsbad, CA, USA, Cat. No. L3000015) and RNAi Max reagent (Invitrogen, Cat. No. 13778150), respectively, according to the manufacturer’s instructions. The empty vector (EV) and pcDNA3.1-AEG-1-HA plasmids were provided by Prof. Seok-Geun. Lee (Kyung Hee University, Seoul, Korea). The siRNAs used for this study were obtained from Genolution Inc. (Seoul, Korea). The siRNA duplex sequences used in this study are as follows: siCTL: sense: 5′-ACUCUAUCUGCACGCUGACUU-3′; antisense: 5′-GUCAGCGUGCAGAUAGAGUUU-3′. siNCL #1: sense: 5′-UCCAAGGUAACUUUAUUUCUU-3′; antisense: 5′-GAAAUAAAGUUACCUUGGAUU-3′. siNCL #2: sense: 5′-UUCUUUGACAGGCUCUUCCUU-3′; antisense: 5′-GGAAGAGCCUGUCAAAGAAUU-3′.

### 2.3. Western Blotting

Cell extracts were prepared with NETN lysis buffer (0.5% NP-40, 50 mM Tris-HCl [pH 8.0], 150 mM NaCl, and 1 mM EDTA) supplemented with a protease (GeneDepot, Cat. No. 3100-01) and phosphatase (GeneDepot, Cat. No. 3200-01) inhibitor cocktail. Protein concentrations were measured via the Bradford assay using a microplate reader (Promega, Madison, WI, USA, Cat. No. GM3000). The protein samples were separated on 8% SDS-PAGE gels and then transferred to nitrocellulose membranes. The membrane was blocked in 5% skim milk in TBST and incubated with the primary antibodies listed below. After washing with TBST, the membrane was incubated with HRP-conjugated secondary antibodies at room temperature. The Fusion Solo Chemidoc system (Vilber, Marne-La-Vallée, Collégien, France) was used to detect chemiluminescence. Protein band intensities were measured using the Image J software (ver. 1.53a, National Institutes of Health, Bethesda, MD, USA). Original western blots can be found at Figure S7.

### 2.4. Antibodies

Anti-c-Met (#8198), anti-AEG-1 (#9596), anti-NCL (#14574), anti-phospho-c-Met (Tyr 1234/1235; #3077), and rabbit IgG isotype control (#3900) antibodies were purchased from Cell Signaling Technology. Anti-β-actin (#AM4302) and anti-HA (#26183) antibodies were purchased from Invitrogen. The HRP-conjugated secondary antibodies (#31430, #31460) were also purchased from Invitrogen.

### 2.5. Interactome Profiling

293T cells were transfected with EV or pcDNA 3.1-AEG-1-HA. For the immunoprecipitation of AEG-1, anti-HA antibodies (Invitrogen, Cat. No. 26183) were added to cell lysates and incubated at 4 °C overnight. Then, protein A/G agarose beads (Santa Cruz Biotechnology, Dallas, TX, USA, Cat. No. sc-2003) were added and incubated at 4 °C for 4 h with gentle rocking. The beads were then washed 5 times with NETN lysis buffer. The immunoprecipitated AEG-1 protein complex was in-gel digested, followed by LC-MS/MS analysis as described previously [23]. The raw MS data were analyzed and visualized via Maxquant (ver. 1.5.3.8, Max Planck Institute of Biochemistry, Martinsried, Germany) and Perseus software (ver. 1.6.14.0, Max Planck Institute of Biochemistry, Martinsried, Germany).

### 2.6. In-Gel Digestion

Samples were briefly separated in SDS-PAGE and coomassie blue stained before being destained with 10% MeOH mixed with 5% acetic acid overnight. Gel fragments were then sliced and washed with destaining buffer (50 mM AMBIC buffer, 50% MeOH) three times. Afterwards, samples were reduced and alkylated via treatment with reduction buffer (50 mM AMBIC buffer, TCEP 2 mM) at 37 °C for 15 min and alkylation buffer (50 mM AMBIC buffer, 20 mM IAA) at room temperature for 15 min. Then, the samples were digested by using trypsin (200 ng) at 37 °C overnight and were eluted with elution buffer (50% ACN, 0.1% TFA).

### 2.7. Co-Immunoprecipitation (Co-IP)

Cell lysates were incubated with anti-HA and anti-NCL antibodies at 4 °C overnight with gentle rocking. Then, the samples were incubated with TrueBlot anti-rabbit Ig IP beads (Rockland, Gilbertsville, PA, USA, Cat. No. 88-1688-31) at 4 °C for 4 h with gentle rocking before being boiled for 5 min in SDS sample loading buffer (Bio-Rad, Hercules, CA, USA, Cat. No. 1610747) containing 2-mercaptoethanol (Bio-Rad, Cat. No. 1610710).

### 2.8. Wound-Healing Assay

Wounds were created using silicon inserts (ibidi, Gräfelfing, Germany, Cat. No. 80469) according to the manufacturer’s instructions. Briefly, inserts were placed on 6-well plates and cells were seeded to generate a confluent monolayer. After 24 h of incubation, inserts and floating cells were removed. Image of wound closure was captured at 0, 24, and 30 h using a fluorescence microscope.

### 2.9. Transwell Migration & Invasion Assay

The cell migration and invasion assays were conducted using a transwell chamber system (Corning, Corning, NY, USA, Cat. No. 3422). Briefly, cells were first washed with PBS twice and resuspended in serum-free media. The upper chamber was then placed in 24-well plates containing culture media with 10% FBS, and cells were seeded on the upper chamber. In the case of the invasion assay, the upper chamber was pre-coated with matrigel. After incubating for 24–48 h, cells were fixed with 4% paraformaldehyde (PFA) and stained with 0.25% crystal violet. Cells in the upper chamber were removed by cotton swab and washed with water twice. Images of the cells in the lower chamber were then captured using a fluorescence microscope.

### 2.10. Colony Formation (Clonogenic) Assay

Cells were seeded into 6-well plates and incubated at 37 °C for eight days while changing the medium every three days. After incubation, colonies were fixed with 4% PFA and stained with 0.25% crystal violet.

### 2.11. Phospho-Tyrosine RTK Array

Tyrosine phosphorylations of receptor tyrosine kinases were analyzed via the Proteome Profiler Human Phospho-RTK Array Kit (R&D Systems, Cat. No. ARY001B) according to the manufacturer’s instructions. Briefly, the array membrane was blocked with array buffer 1, treated with 300 μg of lysate, and then incubated at 4 °C overnight. After washing, the membrane was incubated with HRP-conjugated anti-phospho-tyrosine antibodies at room temperature for 2 h. The Fusion Solo Chemidoc system (Vilber, Marne-La-Vallée, Collégien, France) was used to detect chemiluminescence.

### 2.12. Statistical Analysis

The results are expressed as mean ± standard error (SE). The significance of the results was determined by Student *t*-test (2-tailed) using Microsoft EXCEL (Microsoft, Inc., Redmond, WA, USA).

## 3. Results

### 3.1. Identification of NCL as a Novel AEG-1-Interacting Protein

**Figure 1:**
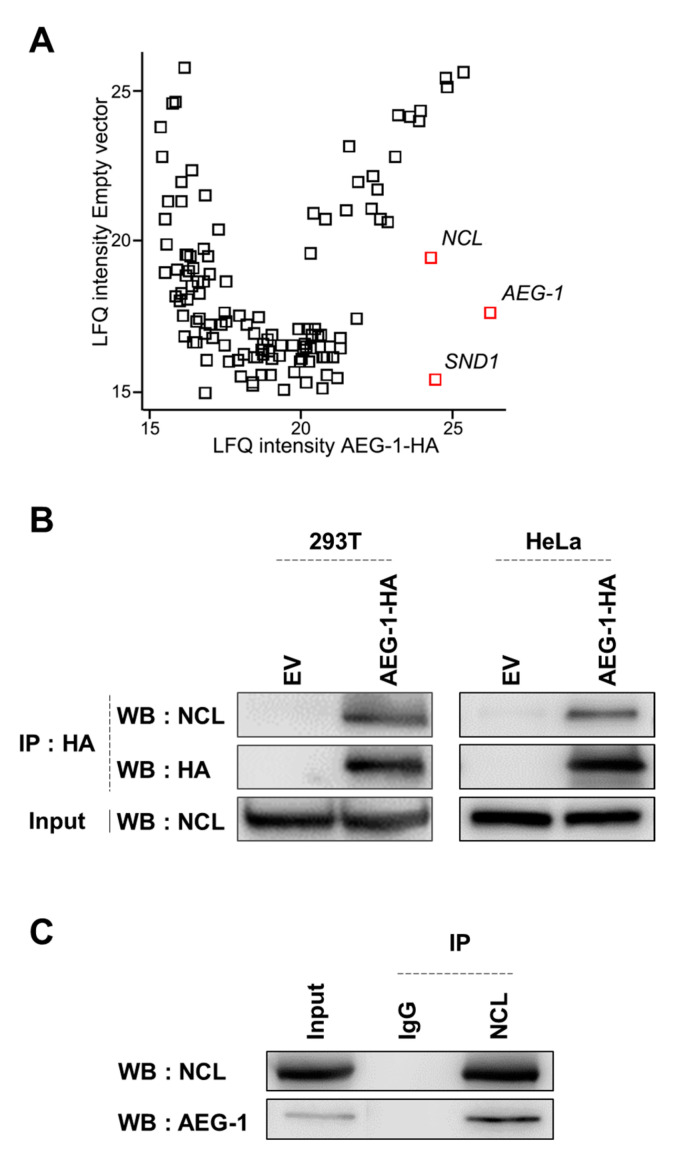
Identification of NCL as a novel AEG-1-interacting protein. (**A**) The scatter plot shows label free quantification (LFQ) values of proteins identified from AEG-1-HA (X-axis) or empty vector (Y-axis) transfected samples. Perseus software (ver. 1.6.14.0) was employed for visualization. (**B**) 293T and HeLa cells were transfected with either an empty vector (EV) or the AEG-1-HA expression vector. Immunoprecipitation (IP) was performed using anti-HA antibodies; co-immunoprecipitated NCL was analyzed by western blot. (**C**) Endogenous NCL was immunoprecipitated in 293T cells and co-immunoprecipitated AEG-1 was analyzed by western blot.

To identify novel interacting proteins, we mapped the astrocyte elevated gene-1 (AEG-1) interactome via affinity purification followed by LC-MS/MS. HA-tagged AEG-1 was expressed in 293T cells before whole cell lysates were subjected to immunoprecipitation (IP) with agarose immobilized anti-HA antibodies. The mass spectrometry analysis revealed various proteins in the AEG-1 protein complex (Table 1). To filter out false-positive hits, we constructed a scatter plot using Perseus software (ver. 1.6.14.0), which resulted in three filtered hits; AEG-1 (bait), SND1, and nucleolin (NCL) (Figure 1A). Of note, SND1 was previously identified as an AEG-1 interacting protein and is associated with the metastasis promoting function of AEG-1 [17]. The analysis led us to hypothesize that NCL was a novel AEG-1 interacting protein. To validate the interaction between AEG-1 and NCL, we performed co-IP experiments in both 293T and HeLa cells. We found that endogenous NCL did interact with HA-tagged AEG-1 in these two cell lines (Figure 1B). Further, the interaction between endogenous AEG-1 and NCL was confirmed by co-IP experiments in 293T cells (Figure 1C). Taken together, the results indicate that NCL is a novel AEG-1-interacting protein.

### 3.2. Silencing NCL Markedly Decreased AEG-1 Induced HeLa Cell Migration

**Figure 2:**
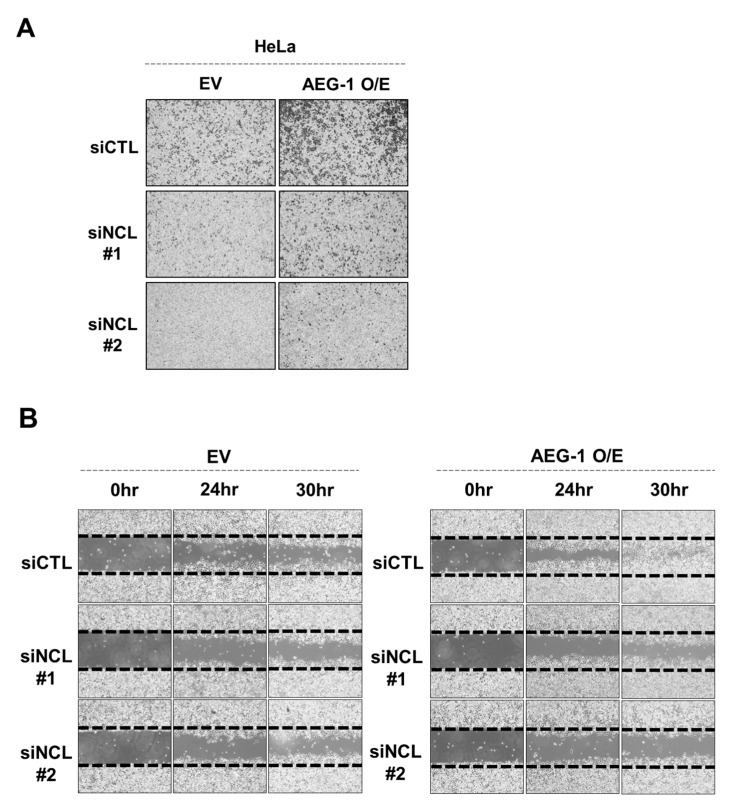
Silencing NCL markedly decreased the migration induced by AEG-1 in HeLa cells. Cells were transfected with indicated siRNAs before the AEG-1-HA or empty vector (EV) plasmids were transfected two days later. Cell migration was analyzed by transwell-based migration assay (**A**) or wound healing assay (**B**). Representative data from three independent experiments with similar results are shown. Validation of transfection and quantitative analyses are shown in Figure S1. O/E: over expression.

NCL belongs to a family of RNA binding proteins and is important for cell growth and proliferation via multiple mechanisms including ribosome biosynthesis, rRNA transcription, and pre-RNA processing [24]. NCL was originally identified as a nuclear protein, however, additional studies have shown that it is also expressed on the cell surface. This is especially the case in cancer cells, and targeting cell surface NCL has been shown to suppress tumor growth [25,26,27]. Currently, the molecular mechanism by which NCL promotes tumorigenesis is not fully elucidated. Here, we hypothesized that NCL could be important for the oncogenic function of AEG-1. To test this, we examined whether NCL knockdown would impair the cancer metastasis induced by the ectopic expression of AEG-1. The transwell migration and wound-healing assay revealed that the cell migration induced by ectopic AEG-1 expression was significantly impaired when NCL was silenced in HeLa cells (Figure 2A,B). These results suggest that NCL could be a novel mediator of the oncogenic function of AEG-1.

### 3.3. AEG-1 Is Aberrantly Expressed in Breast Cancer and Is Associated with a Poor Prognosis

To decide the most suitable cancer model for the functional study of the AEG-1-NCL protein complex, we investigated genetic aberrations of AEG-1 in human cancer tissues employing public databases. The cancer genome atlas (TCGA) database showed that amplification of AEG-1 is the most common alteration in numerous human cancer tissues (Figure S2A). Next, we analyzed the AEG-1 expression levels in normal tissues and five different cancer types; Bladder urothelial carcinoma (BLCA), Breast invasive carcinoma (BRCA), Ovarian serous cystadenocarcinoma (OV), Liver hepatocellular carcinoma (LIHC), and Prostate adenocarcinoma (PRAD), all of which have been shown to frequently have amplified AEG-1 (Figure S2A). The gene expression profiling interactive analysis (GEPIA) database revealed the expression of AEG-1 was pronouncedly elevated in breast cancer tissues compared to other cancer types (Figure S2B). The Oncomine database also showed that the expression of AEG-1 was markedly elevated in tumors compared to normal breast tissues (Figure S2C). Of note, the GENT2 database [28] indicated that the expression of AEG-1 was elevated in triple negative breast cancer (TNBC) compared to luminal A breast cancer, which is less aggressive than TNBC, suggesting AEG-1 expression is associated with breast cancer aggressiveness (Figure S2D). Furthermore, the overall survival (OS) and disease free survival (DFS) of breast cancer patients with high AEG-1 expression was significantly decreased according to GEPIA and Kaplan Meier (KM)-plotter database (Figure S2E,F). Collectively, these analyses indicate that breast cancer could be the most suitable model system to investigate the role of the novel AEG-1 interacting protein, NCL.

### 3.4. Silencing NCL Reduces AEG-1 Induced Proliferation, Migration, and Invasion in Breast Cancer

**Figure 3:**
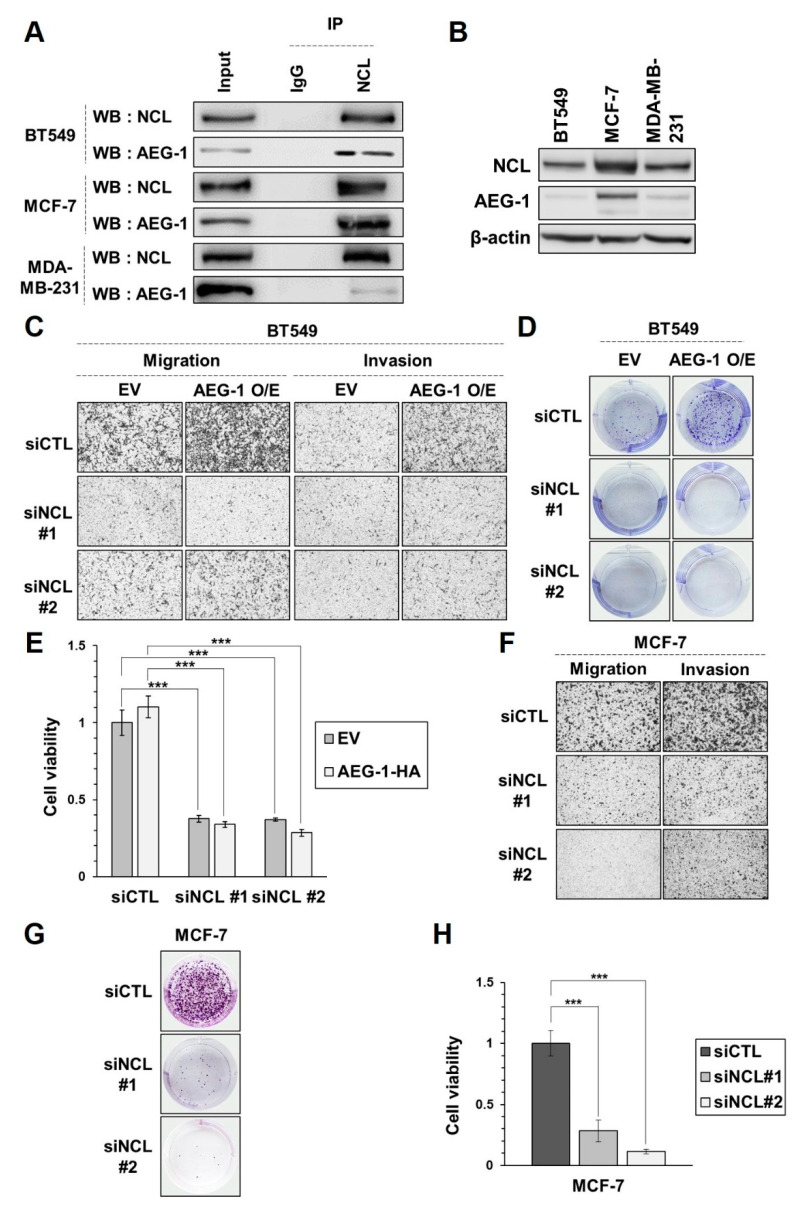
NCL knockdown reduces proliferation as well as the cell migration/invasion induced by the overexpression of AEG-1 in breast cancer cell lines. (**A**) Endogenous NCL was immunoprecipitated in a panel of breast cancer cell lines (BT549, MCF-7, MDA-MB-231), then co-immunoprecipitated AEG-1 was detected by western blot. (**B**) The expression levels of AEG-1 and NCL were analyzed in breast cancer cell lines. (**C**–**E**) BT549 cells were transfected with the indicated siRNAs before the AEG-1-HA or empty vector (EV) plasmids were transfected 2 days later. Migration/invasion and proliferation of transfected cells were analyzed by transwell migration/invasion assay (**C**), and clonogenic (**D**) and MTT (**E**) assay, respectively. (**F**–**H**) MCF-7 cells were transfected with the indicated siRNAs, then the migration/invasion and proliferation of the transfected cells were analyzed by transwell migration/invasion assay (**F**), and clonogenic (**G**) and MTT (**H**) assay, respectively. Representative data from three independent experiments with similar results are shown. *, *p* < 0.05; **, *p* < 0.01; and ***, *p* < 0.001 with mean ± SE are shown. Validation of transfection and quantitative analyses are shown in Figure S3. O/E: over expression.

Previous studies have shown that AEG-1 promotes the proliferation, migration, and invasion of cancer cells, and is associated with a poor prognosis in breast cancer [19,20,21,22]. As previously shown in HeLa cells, interactions between the endogenous AEG-1 and NCL were also detected in three breast cancer cell lines (Figure 3A). We examined the expression level of AEG-1 and NCL in a panel of breast cancer cell lines, and observed that both AEG-1 and NCL were highly expressed in MCF-7 compared to other breast cancer cell lines (Figure 3B). Next, we tested whether NCL is important for the tumorigenic function of AEG-1 in breast cancer cells. To that end, we overexpressed AEG-1 in BT549, where AEG-1 expression was shown to be marginal. We found that cell migration, invasion, and proliferation were elevated by AEG-1, and that silencing NCL could abrogate the enhanced cancer cell growth and motility induced by the ectopic expression of AEG-1 (Figure 3C–E). Further, we examined the effect of NCL silencing in breast cancer cells where AEG-1 was already expressed abundantly. We found that cell migration, invasion, and proliferation were impaired by NCL knockdown in MCF-7 cells (Figure 3F–H). These results indicate that NCL plays an important role in AEG-1-mediated tumorigenesis in breast cancer.

### 3.5. NCL Regulates the Signal of AEG-1 by Reducing the Phosphorylation of C-Met

**Figure 4:**
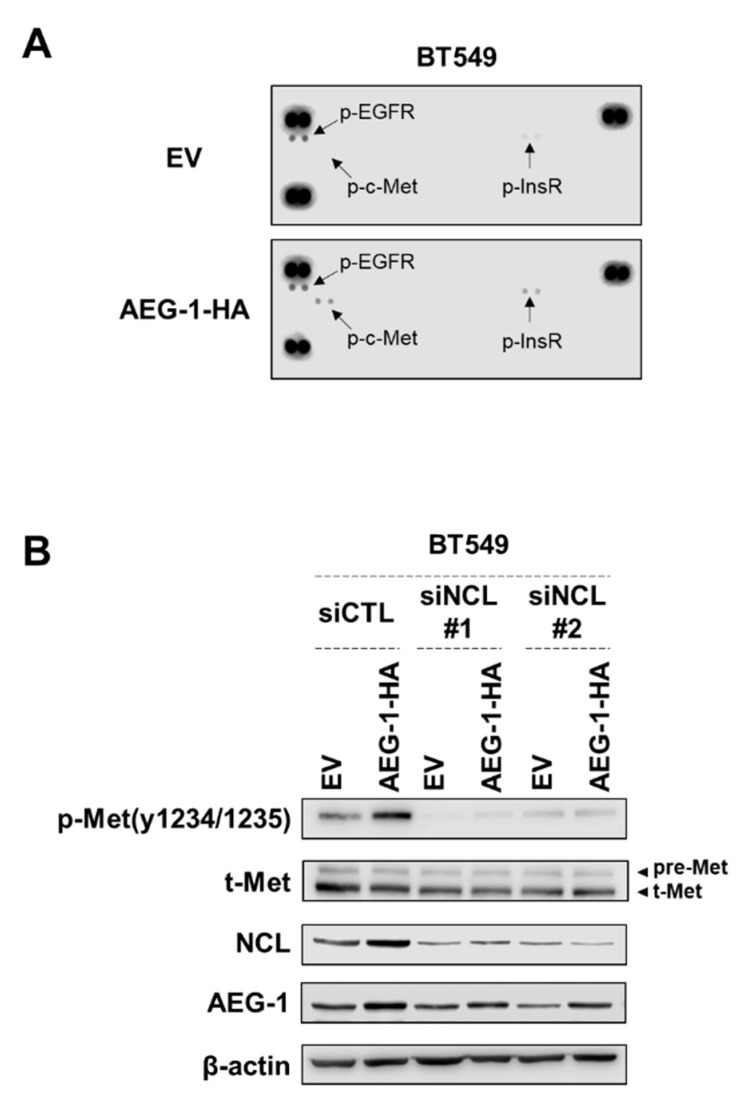
AEG-1 promotes c-Met activating tyrosine phosphorylation and NCL knockdown reduces AEG-1-induced c-Met activation in breast cancer cells. (**A**) BT549 cells were transfected with the AEG-1-HA or empty vector (EV) plasmids. The global tyrosine phosphorylation of receptor tyrosine kinases was then analyzed by Human Phospho-Receptor Tyrosine Kinase (RTK) Array Kit. (**B**) BT549 cells were transfected with the indicated siRNAs, then AEG-1-HA or empty vector plasmids were transfected two days later. Cell lysates were subjected to western blotting with the indicated antibodies. Representative data from three independent experiments with similar results are shown. Quantitative analyses are shown in Figure S6. p-Met: phospho-Met, t-Met: total-Met, pre-Met: precursor-Met.

Next, we attempted to identify the molecular mechanisms by which NCL regulates AEG-1 function. Previous studies have shown that AEG-1 promotes multiple oncogenic signaling pathways including the PI3K/AKT, NF-κB, and MAPK pathways [11,12,13,18,29]. However, we did not observe any evidence that the phosphorylation of p65, IκBα, AKT, and ERK were affected by either AEG-1 overexpression or NCL knockdown in both HeLa and breast cancer cells (Figure S4). Similarly, NF-κB-dependent reporter gene activity and the major NF-κB target gene expression (IL-8) were also not affected (Figure S5). These results raise the possibility that the AEG-1-NCL complex could induce novel oncogenic signaling pathways. Not only is NCL specifically expressed on the cell surface of cancer cells [30,31,32,33], but it is also known to regulate cancer signals through interactions with ErbB family proteins [34,35,36,37]. Therefore, we hypothesized that the interaction between AEG-1 and NCL could promote receptor tyrosine kinase (RTK) activity in breast cancer tissues. To test this possibility, we conducted a phospho-RTK array to identify the level of RTK activation caused by the ectopic expression of AEG-1. We found that tyrosine phosphorylations of c-Met and InsR were significantly elevated by AEG-1 (Figure 4A). Given the well-established function of c-Met in the oncogenic signaling pathways [38,39,40], we focused on c-Met for the validation experiments. We confirmed that activating c-Met tyrosine phosphorylation was elevated by the ectopic expression of AEG-1. Interestingly, silencing NCL could abrogate c-Met tyrosine phosphorylation (Figure 4B). These results suggest that the AEG-1-NCL complex promotes cancer growth and invasion, potentially through promoting c-Met activation.

## 4. Discussion

The expression of AEG-1 is often deregulated in solid tumors [41] and is closely linked to multiple oncogenic signaling pathways, including the Ha-Ras, PI3K/AKT, NF-κB, c-Myc, and Wnt/β-catenin pathways [11,12,18,29]. Protein-protein interaction mapping is important for understanding the oncogenic function of AEG-1 since AEG-1 interacts with various proteins including c-Jun, p300, NF-κB, IKKβ, AKT2, and SND1 to activate oncogenic signaling pathways [13,14,15,16,17]. Here, we report NCL as a novel AEG-1 interacting protein. NCL is known as a nuclear phosphoprotein and is involved with many functions including pre-rRNA transcription, ribosome assembly, chromatin structure, and rDNA transcription [24,42,43]. Numerous studies have shown that NCL expression is altered in cancers. NCL is upregulated in colorectal, gastric, breast, and lung cancers [32,44,45,46], and targeting NCL using aptamers was shown to reduce the invasiveness of breast cancer cells [45]. The mechanisms by which NCL promotes cancer are diverse [33]. High levels of NCL in cancer cells is believed to promote tumorigenesis by facilitating ribosomal RNA synthesis, which can support protein synthesis in rapidly proliferating cells [47]. NCL reportedly controls the expression of a panel of oncogenes including VEGF [48], IL-9R [49], and HPV18 [50]. In addition, cell surface NCL interacts with ErbB1 [35] and Ras [51], promoting RTK-driven Ras signaling. In future, it would be worthwhile to investigate how AEG-1 is linked to enhanced rRNA metabolism and oncogenic signaling in cancer cells driven by aberrant NCL expression.

Our results show that silencing NCL could impair the oncogenic function of AEG-1. Given its diverse roles in multiple cancer signaling pathways, our study provides evidence suggesting that NCL could be involved in AEG-1-mediated oncogenic mechanisms, such as the NF-κB or AKT pathways. Unfortunately, in breast cancer cells, we could not observe how the ectopic expression of AEG-1 activated those signaling molecules, and thus we were unable to examine how NCL regulates AEG-1-mediated signaling pathways. Of note, we discovered that the ectopic expression of AEG-1 could promote c-Met activation in breast cancer, and that knockdown of NCL could abrogate that effect. The mechanisms by which AEG-1 induces c-Met tyrosine phosphorylation is not clear. c-Met mRNA expression was reportedly induced by AEG-1 [22], however we could not observe total-c-Met protein expression was induced by AEG-1 overexpression in BT549 cells. Of note, it has recently been reported that NCL interacts with c-Met in gastric cancer cells and combinational inhibition of these two proteins could lead to anti-cancer efficacy [52]. Given that NCL-ErbB1interaction promotes ErbB1 dimerization and activation [34], NCL possibly induces c-Met activation as well in the presence of AEG-1. Another possibility is that AEG-1 could inhibit the function of protein-tyrosine phosphatases (PTP) targeting c-Met. DEP-1 is reportedly a tyrosine phosphatase that negatively regulates c-Met [53], thus it may be worth investigating the possibility that AEG-1 regulates DEP-1 through interactions with NCL.

In summary, our results show that NCL is a novel interacting partner of AEG-1. Although AEG-1 is a *bona fide* oncogene, therapeutic approaches directly targeting AEG-1 remain in their infancy. Development of protein-protein interaction inhibitors that disrupt the AEG-1-NCL complex could be a promising approach. Alternatively, an aptamer-based targeting of NCL, which has garnered significant attention of late [25,26,27], may be an attractive approach for the treatment of breast cancers with aberrant AEG-1 expression.

## 5. Conclusions

Our results clearly show that NCL is a novel, AEG-1 interacting protein. The AEG-1-NCL protein complex promotes the proliferation, migration, and invasion of breast cancer cells, potentially via mediation of c-Met activation. Our study suggests that targeting the AEG-1-NCL protein complex could be an effective therapeutic approach for the treatment of some breast cancers.

## Figures and Tables

**Table 1:** List of AEG-1 interacting proteins revealed by LC-MS/MS analysis.

Uniprot ID	Protein Name	Peptide Count
Q86UE4	LYRIC	25
Q7KZF4	SND1	17
P19338	NCL	19
Q02878	RL6	5
Q9UQ80	PA2G4	4
P18124	RL7	3
P47914	RL29	2
P07900	HS90A	3
Q15233	NONO	4

## Data Availability

The data that support the findings of this study are available from the corresponding author’s e-mail upon reasonable request.

## Supplementary Materials

The following are available online at https://www.mdpi.com/article/10.3390/cancers13112842/s1, Figure S1: Supporting data for Figure 2, Figure S2: AEG-1 is aberrantly expressed in breast cancer and is associated with a poor prognosis, Figure S3: Supporting data for Figure 3, Figure S4: Analysis of AEG-1 downstream phosphoproteins, Figure S5: Analysis of NF-κB activity in cells transfected with AEG-1-HA and siNCL, Figure S6: Supporting data for Figure 4. Figure S7: Full western blots.
