# Supplementary material for: Identification of Nucleolin as a Novel AEG-1-Interacting Protein in Breast Cancer via Interactome Profiling
Source: Cancers (Basel). 2021 Jun 7;13(11):2842. doi: 10.3390/cancers13112842 (PMC8201222; doi:10.3390/cancers13112842)
Supplement: Supplementary file 1 [file cancers-13-02842-s001.zip › cancers-1211279-SI/cancers 1211279_supplementary figure.pdf]

Supplementary materials

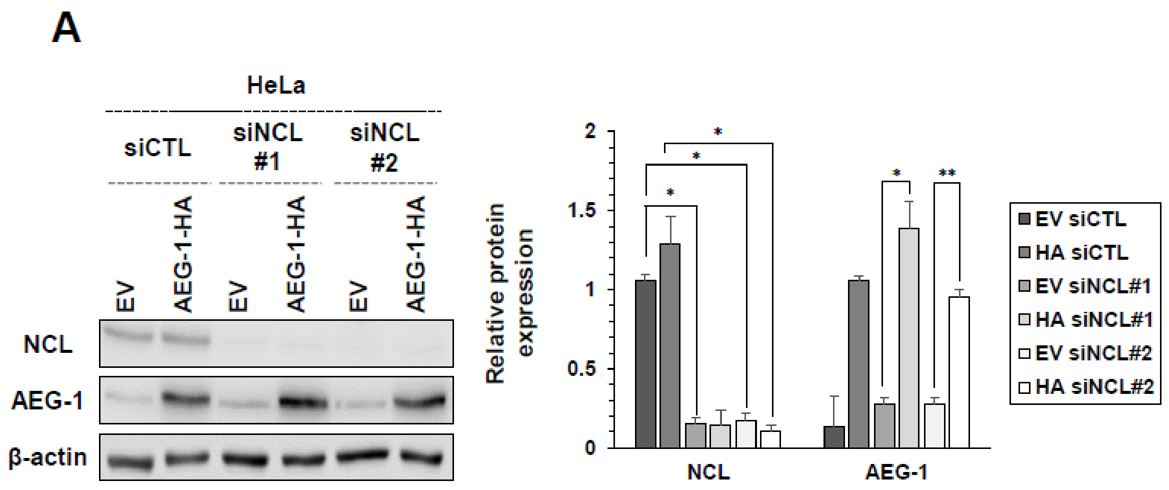

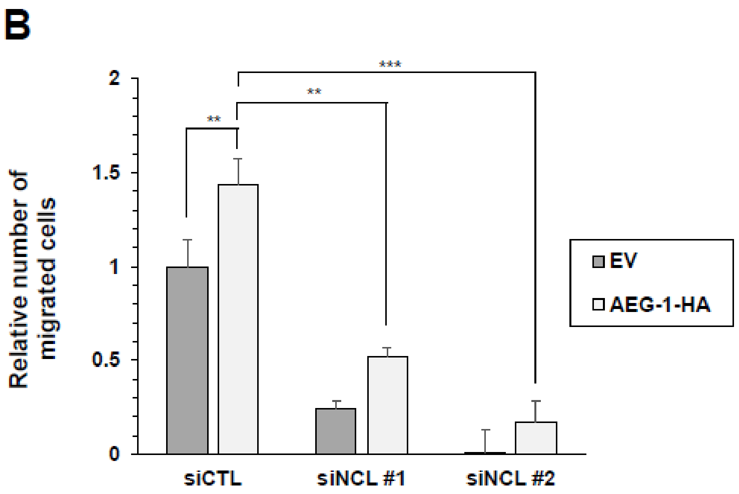

Figure S1. Supporting data for figure 2. (A) AEG-1 overexpression and NCL knockdown in figure 2 were validated by western blot. Relative band intensities normalized by β-actin are shown below. EV: empty vector. HA: AEG-1-HA. (B) Quantification of transwell migration assay results presented in figure 2A. Average cell numbers counted from 5 independent microscopic fields are shown. *, $P < 0.05$; **, $P < 0.01$; and ***, $P < 0.001$ with mean ± SE are shown.

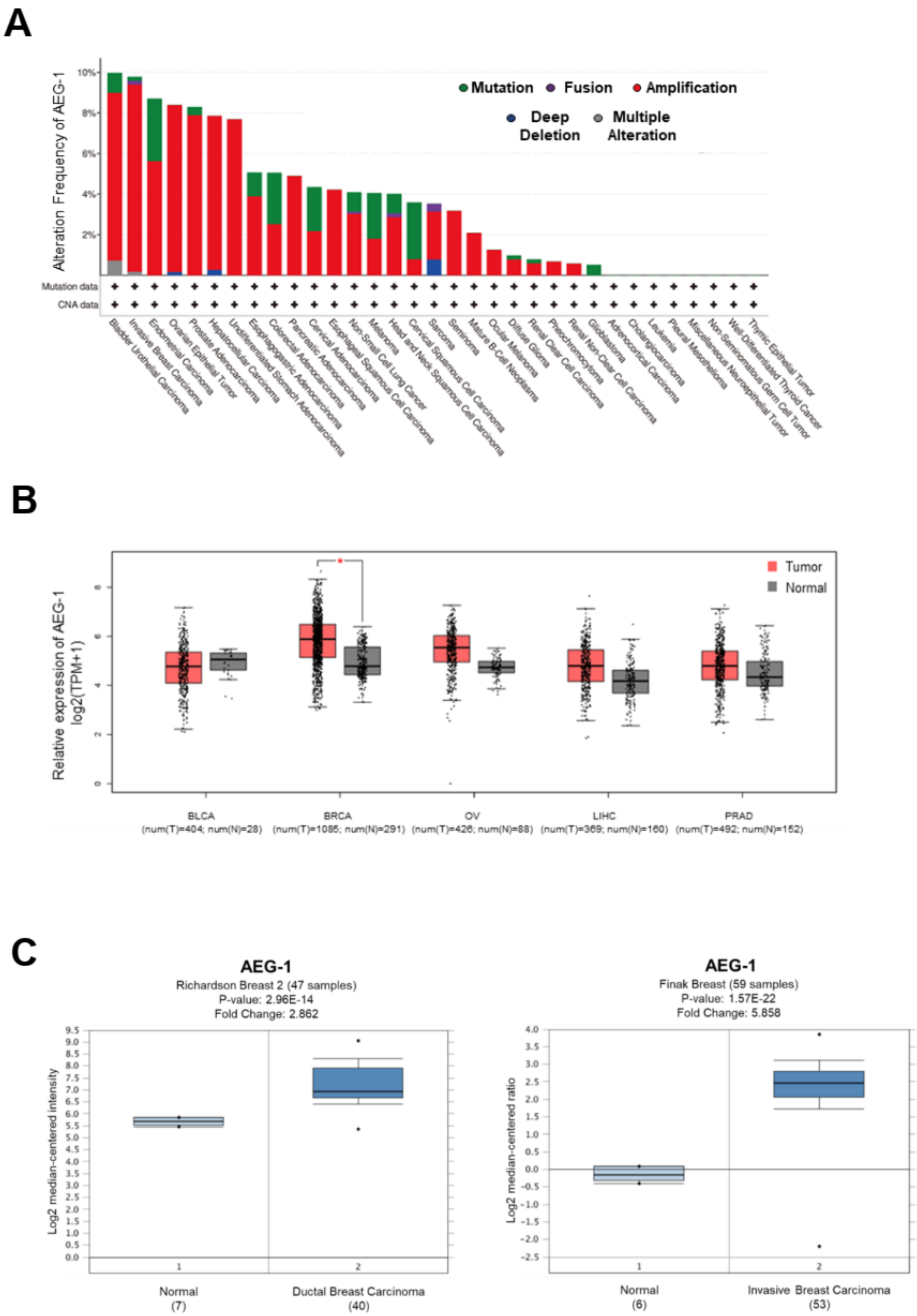
A
Alteration Frequency of AEG-1
Mutation
Fusion
Amplification
Deep Deletion
Multiple Alteration
Mutation data
CNA data
B
Relative expression of AEG-1 log2(TPM+1)
Tumor
Normal
BLCA (num(T)=404; num(N)=28)
BRCA (num(T)=1085; num(N)=291)
OV (num(T)=426; num(N)=88)
LIHC (num(T)=369; num(N)=160)
PRAD (num(T)=492; num(N)=152)
C
AEG-1
Richardson Breast 2 (47 samples)
P-value: 2.96E-14
Fold Change: 2.862
Log2 median-centered intensity
Normal (7)
Ductal Breast Carcinoma (40)
AEG-1
Finak Breast (59 samples)
P-value: 1.57E-22
Fold Change: 5.858
Log2 median-centered ratio
Normal (6)
Invasive Breast Carcinoma (53)

D

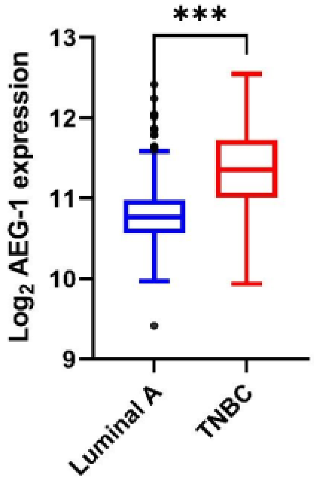
***
Log2 AEG-1 expression
13
12
11
10
9
Luminal A
TNBC

E

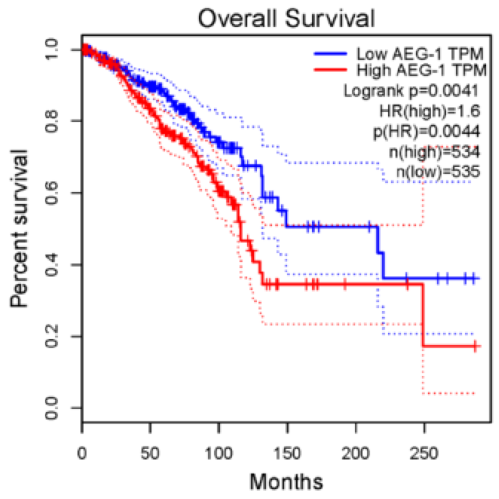
Overall Survival
Low AEG-1 TPM
High AEG-1 TPM
Logrank p=0.0041
HR(high)=1.6
p(HR)=0.0044
n(high)=534
n(low)=535
Percent survival
0.0
0.2
0.4
0.6
0.8
1.0
0
50
100
150
200
250
Months

F

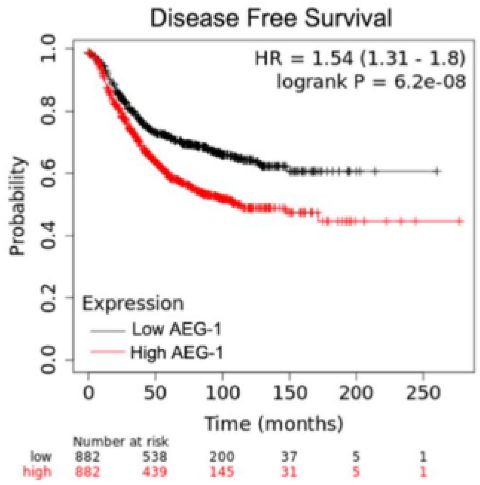
Disease Free Survival
HR = 1.54 (1.31 - 1.8)
logrank P = 6.2e-08
Probability
0.0
0.2
0.4
0.6
0.8
1.0
Expression
Low AEG-1
High AEG-1
0
50
100
150
200
250
Time (months)
Number at risk
low 882 538 200 37 5 1
high 882 439 145 31 5 1

Figure S2. AEG-1 is aberrantly expressed in breast cancer and is associated with a poor prognosis. (A) The TCGA database shows multiple abnormalities of the AEG-1 gene present in most cancers, including breast cancer. (B) The GEPIA database shows that AEG-1 protein levels are significantly upregulated in breast cancer tissues compared to normal (|Log2FC| Cutoff: 1, p-value Cutoff: 0.01). (C) The Oncomine database shows that AEG-1 mRNA expression levels are upregulated in breast cancer. (D) The GENT2 database shows AEG-1 expression level was upregulated in TNBC (n=252) than luminal A (n=380) subtype samples ($Log_2FC = 0.558$). *** : $p < 0.001$. (E) The GEPIA database shows that high AEG-1 expression is associated with low overall survival (OS) in breast cancer patients. (F) The KM-plotter database shows that high AEG-1 expression is associated with low disease free survival (DFS) in breast cancer patients.

Figure S3

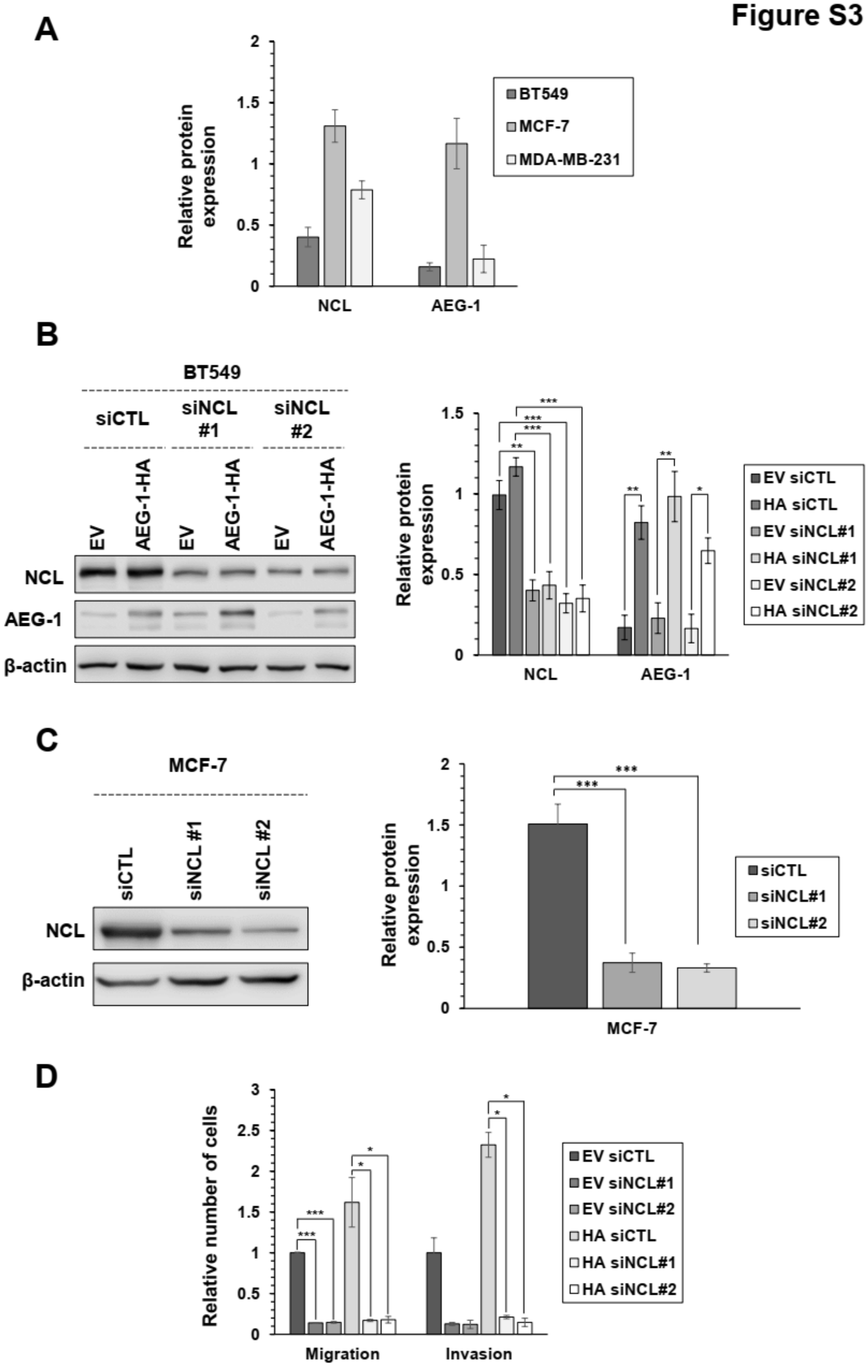
A
Relative protein expression
BT549
MCF-7
MDA-MB-231
NCL
AEG-1
B
BT549
siCTL
siNCL #1
siNCL #2
EV
AEG-1-HA
NCL
AEG-1
β-actin
Relative protein expression
EV siCTL
HA siCTL
EV siNCL#1
HA siNCL#1
EV siNCL#2
HA siNCL#2
C
MCF-7
siCTL
siNCL #1
siNCL #2
NCL
β-actin
Relative protein expression
siCTL
siNCL#1
siNCL#2
D
Relative number of cells
EV siCTL
EV siNCL#1
EV siNCL#2
HA siCTL
HA siNCL#1
HA siNCL#2
Migration
Invasion

E

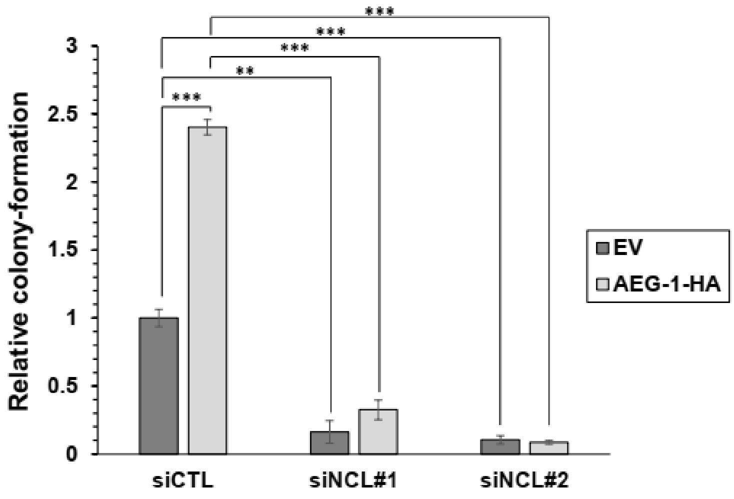

Relative colony-formation
3
2.5
2
1.5
1
0.5
0
***
**
siCTL
siNCL#1
siNCL#2
EV
AEG-1-HA

F

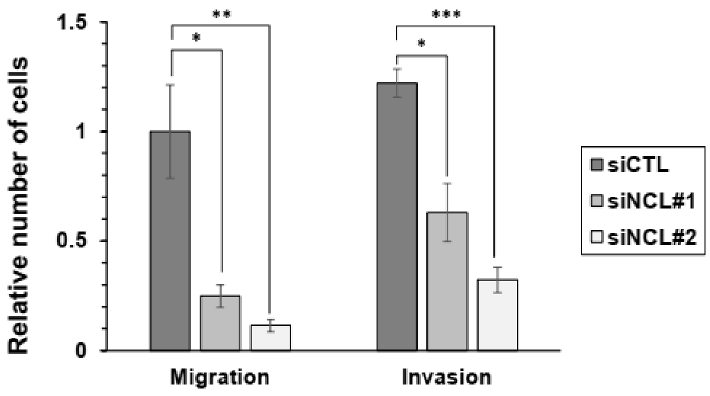

Relative number of cells
1.5
1
0.5
0
*
**
***
Migration
Invasion
siCTL
siNCL#1
siNCL#2

G

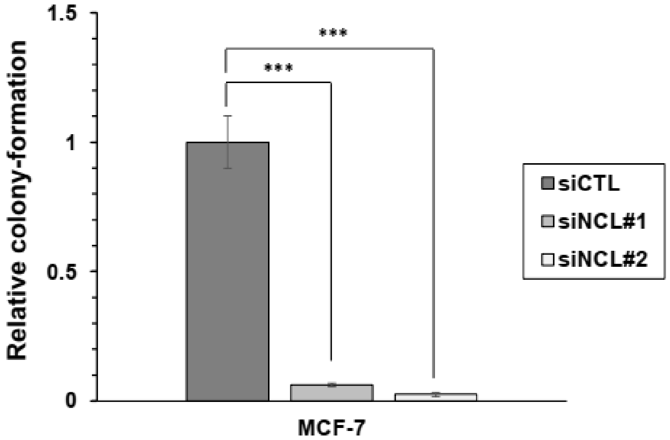

Relative colony-formation
1.5
1
0.5
0
***
MCF-7
siCTL
siNCL#1
siNCL#2

Figure S3. Supporting data for figure 3. (A) Quantification of western blot results presented in figure 3B. Relative band intensities normalized by β-actin are shown. (B and C) Validation of AEG-1 overexpression and NCL knockdown in BT549 (B) and MCF-7 (C) cells by western blot. Relative band intensities normalized by β-actin are shown. EV: empty vector. HA: AEG-1-HA. (D-G) Quantification of results presented in figures 3C (D), 3D (E), 3F (F) and 3G (G). *, P < 0.05; **, P < 0.01; and ***, P < 0.001 with mean ± SE are shown.

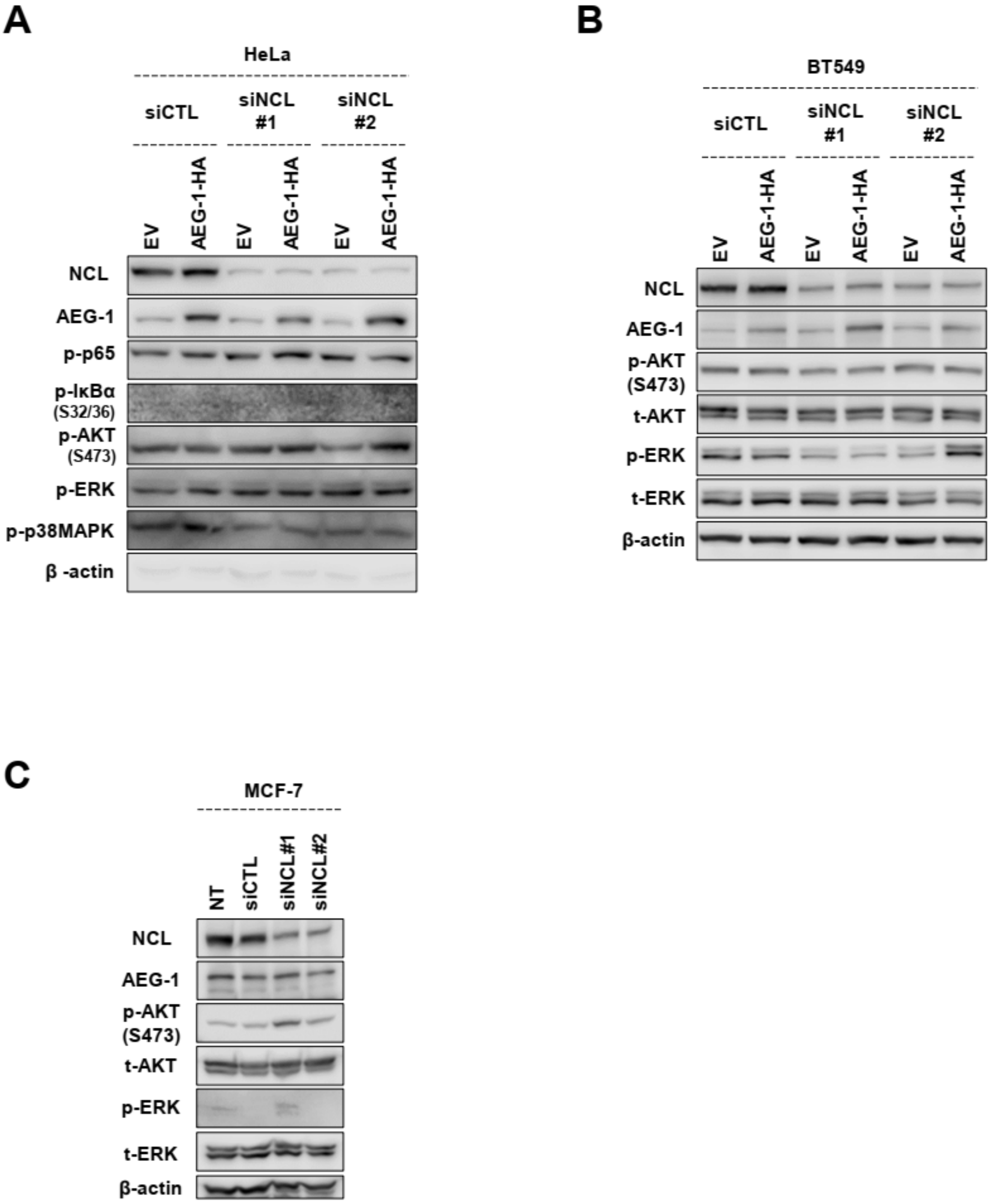

Figure S4. Analysis of AEG-1 downstream phosphoproteins. (A and B) HeLa (A) and BT549 (B) cells were transfected with the indicated siRNAs, then AEG-1-HA or empty vector (EV) plasmids were transfected 2 days later. The phosphorylation of p65, IκBα, AKT, ERK, and p38MAPK was then analyzed by western blot. (C) MCF-7 cells were transfected with siNCL or control siRNA before the phosphorylation of AKT and ERK were analyzed by western blot. *, P < 0.05; **, P < 0.01; and ***, P < 0.001 with mean ± SE are shown.

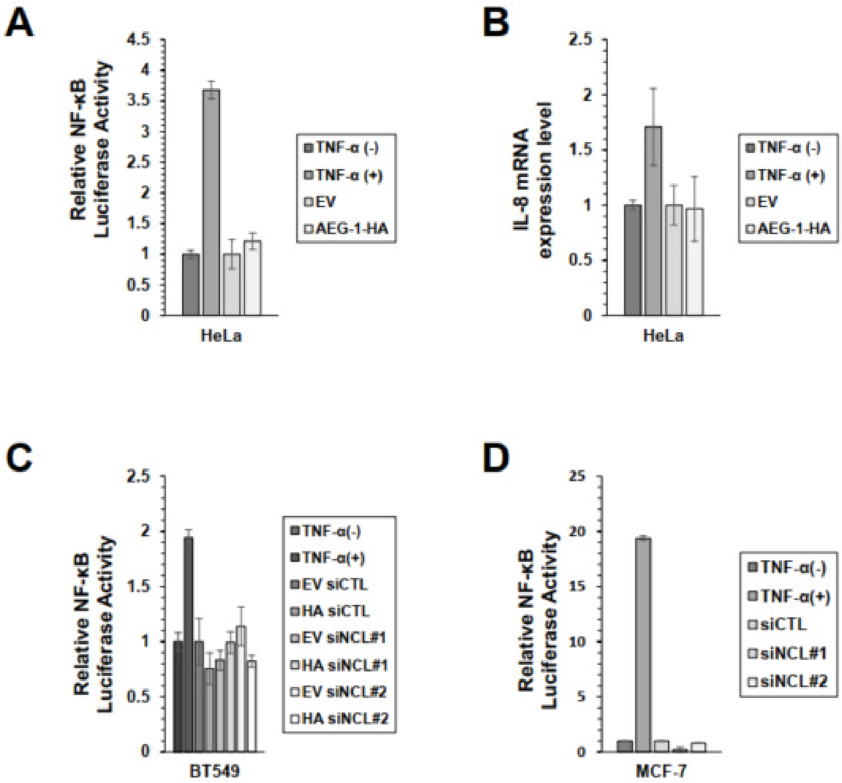

Figure S5. Analysis of NF-κB activity in cells transfected with AEG-1-HA and siNCL. (A and B) NF-κB activation was assessed by luciferase reporter assay (A) and quantitative RT-PCR for human IL-8 (B) in HeLa cells transfected with the AEG-1-HA plasmid. TNF-α treatment was used as a positive control. EV: empty vector. HA: AEG-1-HA. (C and D) NF-κB activation was assessed by luciferase reporter assay in BT549 (C) and MCF-7 (D) cells transfected with the indicated plasmids or siRNAs. *, P < 0.05; **, P < 0.01; and ***, P < 0.001 with mean ± SE are shown.

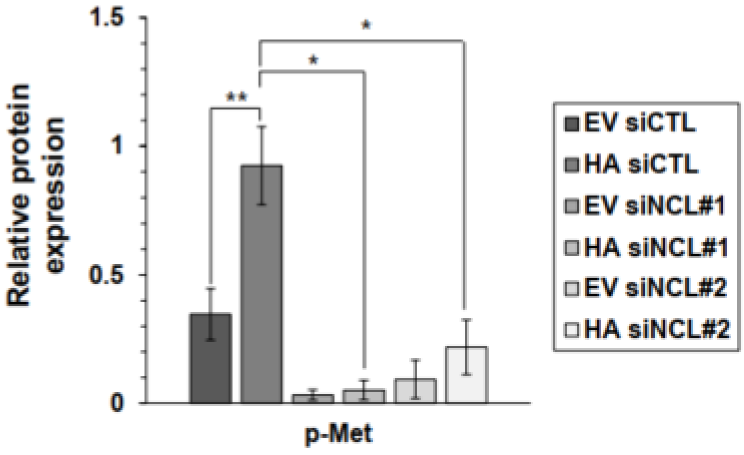

Figure S6. Supporting data for figure 4. Quantification of western blot results presented in figure 4B. Relative band intensities normalized by c-Met are shown. EV: empty vector. HA: AEG-1-HA. *, P < 0.05; **, P < 0.01; and ***, P < 0.001 with mean ± SE are shown.
