# Supplementary material for: Identification of Nucleolin as a Novel AEG-1-Interacting Protein in Breast Cancer via Interactome Profiling
Source: Cancers (Basel). 2021 Jun 7;13(11):2842. doi: 10.3390/cancers13112842 (PMC8201222; doi:10.3390/cancers13112842)
Supplement: Supplementary file 1 [file cancers-13-02842-s001.zip › cancers-1211279-SI/cancers-1211279 Supplementary Materials and Methods.pdf]

Supplementary Materials and Methods

Informatics analysis

The Cancer Genome Atlas (TCGA) (https://www.cbioportal.org) was employed to assess the genetic alterations of AEG-1 in different cancers. The Gene Expression Profiling Interactive Analysis (GEPIA) database (http://gepia.cancer-pku.cn) was employed to assess expression levels of AEG-1 in Bladder urothelial carcinoma (BLCA), Breast invasive carcinoma (BRCA), Ovarian serous cystadenocarcinoma (OV), Liver hepatocellular carcinoma (LIHC), and Prostate adenocarcinoma (PRAD). The GEPIA was also used to analyze the association between AEG-1 expression levels and the overall survival (OS) of breast cancer patients. The Oncomine (http://www.oncomine.org) database was employed to address the relative expression of AEG-1 in normal, ductal breast carcinoma, and invasive breast carcinoma. The Kaplan-Meier (KM) plotter (http://www.kmplot.com) was employed to analyze the association between AEG-1 expression and the disease free survival (DFS) of breast cancer patients. The GENT2 database (http://gent2.appex.kr/gent2) was employed to assess differential AEG-1 expression between luminal A and triple negative breast cancer (TNBC), and Prism 9 software (Prism 9, GraphPad, La Jolla, CA, USA) was used for data visualization.

Antibodies

Anti-AKT (#4691), anti-ERK (#4695), anti-c-Met (#8198), anti-AEG-1 (#9596), anti-NCL (#14574), anti-phospho-p65 (#3033), anti-phospho-c-Met (Tyr 1234/1235; #3077), anti-phospho-AKT (Ser 473; #4051), anti-phospho-ERK (#4370), anti-phospho-p38MAPK (#9211) and anti-phospho-IκBα (Ser 32/36; #9246) antibodies were purchased from Cell Signaling Technology. Anti-β-actin (AM4302) antibodies were purchased from Invitrogen. The HRP-conjugated secondary antibodies (#31430, #31460) were also purchased from Invitrogen.

Real-time qPCR

Total RNA was prepared using an RNA Extraction Kit (Bioneer, Cat. No. K-3140). To obtain cDNA, 400ng of each RNA sample was reverse transcribed using cDNA Master Mix (Cell safe, Cat. No. CDS-200). mRNA levels of human IL-8 were analyzed by real-time PCR using SYBR green (Toyobo, Cat. No. QPK-201). Samples were normalized to the signal generated from glyceraldehyde-3-phosphate dehydrogenase (GAPDH).

Luciferase assay

Cells were plated in 24-well plates and transfected with the indicated reporter gene constructs with a Renilla luciferase construct as an internal control. 48 hours after transfection, luciferase assays were performed using a Dual-luciferase assay system (Promega, Cat. No. E1910) according to the manufacturer's protocol.
